# Influence of Metal Oxide Incorporation on the Structure, Surface Features, and Hydrogen Storage Behavior of ZIF‑8

**DOI:** 10.1021/acsomega.6c01498

**Published:** 2026-07-04

**Authors:** Ayten Ateş, Beyza Evgin, Fulya Karaoğlu

**Affiliations:** Sivas Cumhuriyet University, Faculty of Engineering, Department of Chemical Engineering, Sivas 58140, Turkey

## Abstract

Zeolitic imidazolate framework-8 (ZIF-8), a type of metal–organic framework (MOF), is considered a promising material for cryogenic hydrogen storage due to its high surface area, well-defined microporous structure, and strong thermal stability. In this study, pure ZIF-8 and several composite materials containing iron oxide (Fe_3_O_4_), nickel oxide (NiO), and a combination of both were synthesized using a methanol-assisted method. The aim was to understand how adding these metal oxides affects the structure and hydrogen storage performance. X-ray diffraction and nitrogen adsorption–desorption analyses showed that the characteristic structure of ZIF-8 was preserved in all samples. The pure ZIF-8 had a high surface area of about 2088 m^2^ g^–1^ and a crystallite size of around 43 nm. Hydrogen adsorption measurements under cryogenic conditions revealed a maximum storage capacity of 1.140 wt % for the pure material. The addition of Fe_3_O_4_ and NiO caused slight decreases in hydrogen uptake, which can be explained by partial pore blocking balanced by the formation of new adsorption sites. Increasing the amount of NiO led to a more noticeable reduction in both surface area and hydrogen capacity, whereas Fe_3_O_4_ better maintained the porous structure of ZIF-8. Overall, the results show that hydrogen storage performance depends on both the textural properties and the interactions between the surface and hydrogen molecules, with Fe_3_O_4_ being a more suitable additive than NiO for preserving performance.

## Graphical abstract

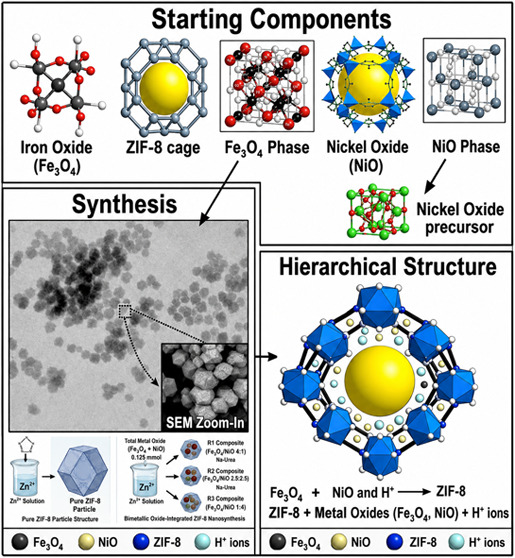

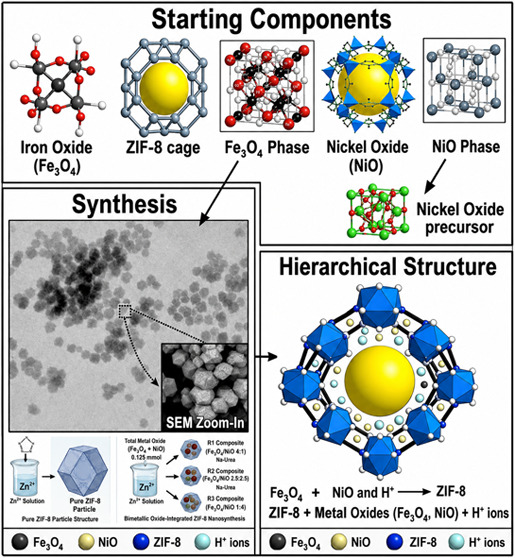

## 1 Introduction

Hydrogen is considered one of the most attractive clean-energy carriers for lowering greenhouse gas emissions. However, its extremely low density and strong flammability make handling and storage difficult. Consequently, creating safe and efficient storage methods is crucial for the progress of hydrogen-powered technologies.^1^

Currently, hydrogen is stored using three primary methods: high-pressure compression, cryo-compression, and material-based storage. Hydrogen is typically stored through high-pressure compression. It involves compressing hydrogen to high pressures, offering rapid filling and release rates without requiring energy input for desorption. However, the energy demand for compression corresponds to roughly 13–18% of hydrogen’s LHV (lower heating value). Moreover, storage containers must be both lightweight and resistant to hydrogen diffusion and embrittlement.^2^

Cryo-compressed hydrogen storage addresses the limitations of both compressed gas and cryogenic liquid hydrogen methods. In this approach, hydrogen is stored as a cryogenic liquid (typically at 20 K) under pressure in insulated containers capable of withstanding both cryogenic temperatures and high pressures. This method allows for increased volumetric hydrogen density—up to 70 g/L at 1 bar and 87 g/L at 240 bar—while minimizing boil-off losses and extending storage times.^3^

Material-based hydrogen storage relies on physisorption and/or chemisorption of hydrogen onto or into solid materials. In physisorption, hydrogen molecules adhere to the surface of porous nanomaterials such as carbon nanotubes or metal–organic frameworks (MOFs), whereas chemisorption involves the dissociation of hydrogen molecules and their integration into the host lattice. While these processes offer safer, lower-pressure alternatives, they are often hindered by slow kinetics, poor reversibility, and the requirement for high desorption temperatures.^4^ Among the various materials explored for material-based hydrogen storage, MOFs have attracted significant interest because they offer adjustable pore structures, exceptionally large surface areas, and well-defined crystallinity. These materials are assembled by coordinating metal ions or metal clusters with organic linkers, resulting in open frameworks known for their stability. As a result of these characteristics, MOFs have been widely adopted as effective platforms for gas storage and separation processes.^5^

Within the metal–organic framework family, zeolitic imidazolate frameworks (ZIF-8) are distinguished by structural motifs inspired by both conventional zeolites and imidazolate coordination frameworks. ZIF-8 has a sodalite (SOD) topology with Zn­(II) centers connected by imidazolate ligands, resulting in a highly stable polyhedral framework. It possesses pore openings of 3.4 Å and internal voids of 11.6 Å, along with high chemical and thermal stability. Notably, ZIF-8 shows promising hydrogen adsorption performance at room temperature, outperforming many conventional MOFs in this regard.^6^

Several studies have investigated ZIFs for hydrogen storage. For instance, Wang et al. synthesized Mg/MOF composites (including Mg/ZIF-8, Mg/ZIF-67, and Mg/MOF-74) via deposition–reduction and showed that porous MOFs could enhance the hydrogen desorption properties of Mg nanoparticles. Particularly, Mg/ZIF-67 exhibited better hydrogenation–dehydrogenation kinetics due to the catalytic role of CoMg_2_ formed in situ, achieving improved cycling stability and lower enthalpy values compared to other Mg/MOF systems.^7^ Similarly, Balderas-Xicohtencatl et al. synthesized ZIF-8 pellets via twin-screw extrusion and reported that pelletization increased packing density without compromising porosity. This approach enabled a high volumetric hydrogen storage capacity (∼35 g/L), close to the theoretical maximum for single crystals, and demonstrated excellent reproducibility and long-term cycling stability under pressure–temperature swing adsorption conditions.^8^

Efforts to improve hydrogen storage kinetics and capacity have also focused on incorporating transition metals into ZIFs. For example, Yang et al. introduced Fe-loaded ZIFs into MgH_2_ and observed significantly improved hydrogenation kinetics and cycling stability, with activation energies for H_2_ adsorption and desorption (27.9 and 104.5 kJ/mol, respectively) notably lower than for many comparable systems. The catalytic effect was attributed to α-Fe derived from Fe-ZIF decomposition, which enhanced interaction with MgH_2_.^9^ Similarly, Shao et al. synthesized Ni/MOF composites using tricarboxybenzene and demonstrated that uniformly dispersed Ni atoms improved hydrogen desorption rates and stability in MgH_2_ systems. The retention of 98.2% capacity after 10 cycles underscored the robustness of the Ni-MOF structure.^10^

Building on these advancements, the primary aim of this research is the preparation and detailed analysis of ZIF-8 and its composites modified with transition metal oxides, specifically Fe_3_O_4_ and NiO, at different molar ratios. ZIF-8 was selected because of its high surface area, chemical and thermal stability, and excellent porosity. Given literature findings indicating degradation of ZIF-8’s crystal structure at metal loadings above 5%,^11^ a 5 mol % metal oxide/ZIF-8 ratio was maintained during synthesis. Composite materials were prepared via a methanol-assisted mixing method. Comprehensive characterization techniques—including XRD, XRF, ICP-MS, XPS, Raman spectroscopy, SEM, STEM, BET, and FTIR—were used to examine the structural, morphological, and chemical features. Hydrogen adsorption–desorption experiments were conducted to evaluate the hydrogen storage capacity of each sample at cryogenic temperatures. The effect of metal oxide incorporation on surface area, pore structure, and hydrogen adsorption performance was systematically investigated, and the optimal metal composition was identified based on adsorption efficiency.

## 2 Experimental Method

### 2.1 Chemicals

All reagents used in this work were of analytical grade and employed without further purification. Zinc nitrate hexahydrate (98%), 2-methylimidazole (99%), N,N-dimethylformamide, methanol (≥99.9%), and iron­(II,III) oxide were obtained from Sigma-Aldrich, while nickel­(II) oxide was purchased from Merck.

### 2.2 Synthesis of Materials

#### 2.2.1 Synthesis of Zeolitic Imidazolate Frameworks (ZIFs)

ZIF-8 was synthesized following a methanol-based procedure adapted from Li et al.^12^ In a typical preparation, zinc nitrate hexahydrate (5.95 g) was dissolved in 100 mL of anhydrous methanol, while a separate solution was prepared by dissolving 2-methylimidazole (6.568 g) in an additional 100 mL of dry methanol. The solutions were stirred individually for approximately 5 min before being combined. The resulting mixture was allowed to react under continuous stirring at 25 °C for 1 h. The resulting solid was separated by centrifuging at 5000 rpm, rinsed with dry methanol, and subsequently dried at 60 °C overnight. The obtained material was labeled ZIF-8.

#### 2.2.2 Synthesis of Metal Oxide/ZIF-8

Composites of ZIF-8 incorporating iron and nickel oxides were prepared by introducing Fe_3_O_4_ and NiO during the framework synthesis. To preserve the structural integrity of ZIF-8, the molar ratio of each metal oxide to zinc nitrate hexahydrate was limited to 5%. For the preparation of the metal oxide/ZIF-8 materials, 2.125 mmol of Zn­(NO_3_)_2_·6H_2_O and 0.125 mmol of the selected metal oxide were dispersed in 20 mL of methanol. A separate solution containing 3.284 g of 2-methylimidazole in 80 mL of methanol was then added. The mixture was stirred thoroughly at ambient temperature for 2 h to allow composite formation. The resulting solids were recovered by repeated methanol washing (three cycles), followed by centrifugation and drying at 60 °C overnight.

For the synthesis of Fe_3_O_4_–NiO/ZIF-8 nanocomposites with different compositions, the total amount of metal oxide was kept constant at 0.125 mmol, while the molar ratios of Fe_3_O_4_ to NiO were adjusted to 4:1 (R1), 2.5:2.5 (R2), and 1:4 (R3). This approach enabled the controlled preparation of bimetallic oxide-incorporated ZIF-8 nanocomposites.

### 2.3 Characterization of Samples

A comprehensive set of analytical techniques was utilized to characterize the structural, morphological, and chemical features of ZIF-8 and metal oxide/ZIF-8 composite materials. Sample morphology, particle dimensions, and elemental distribution were examined by scanning transmission electron microscopy (STEM) and field-emission scanning electron microscopy (FESEM). FESEM images were recorded using a Tescan Mira3 XMU microscope (Brno, Czech Republic)*.* The metal oxide content of the synthesized composites was determined by inductively coupled plasma mass spectrometry (ICP-MS, ICAP Q, Thermo Fisher Scientific) and X-ray fluorescence (XRF) analysis using a Thermo Scientific Niton XL3t GOLDD+ analyzer. For ICP-MS measurements, approximately 5 mg of each sample was completely digested in ultrapure nitric acid (HNO_3_). The resulting solution was subsequently diluted with ultrapure water to the desired volume prior to analysis. The concentrations of Zn and Ni were quantified by ICP-MS, while the Fe content of the composites was quantified by XRF analysis.

Crystallographic structure, phase composition, and framework stability were investigated by X-ray diffraction (XRD) using a Bruker D8 Advance diffractometer equipped with a D/tex Ultra 250 detector and operated with Cu Kα radiation. Functional group vibrations and bonding characteristics were analyzed using Fourier-transform infrared (FTIR) spectroscopy (PerkinElmer Spectrum Two) equipped with a diamond ATR accessory, over the 400–4000 cm^–1^ range. Raman spectra were acquired on a WITec Alpha M+ system employing a 532 nm laser.

Surface elemental composition and chemical states were analyzed by X-ray photoelectron spectroscopy (XPS) using a SpecFlex XPS instrument. Textural properties, including specific surface area and pore structure, were derived from nitrogen adsorption–desorption isotherms collected on an Autosorb 1C analyzer after vacuum degassing of the samples at 80 °C.

### 2.4 Hydrogen Storage Capacity

H_2_ storage capacities of all synthesized samples were determined by hydrogen adsorption–desorption measurements on a Micromeritics 3Flex Surface Characterization Analyzer (version 6.02). Analyses were conducted using H_2_ as the adsorptive gas at a bath temperature of 77 K. Adsorption–desorption isotherms were recorded over an absolute pressure range of 0–0.9 bar, and hydrogen uptake capacities were reported as weight percent H_2_ (wt %).

## 3 Results and Discussion

### 3.1 STEM and XRF Analysis of ZIF-8 and Metal Oxide Composites

Figure 1 presents the STEM images of pristine ZIF-8 and its metal-oxide-modified composites, while the corresponding compositional data are summarized in Table 1. The pure ZIF-8 sample exhibits a well-defined polyhedral morphology, confirming the successful formation of the framework. This structural uniformity is attributed to the use of methanol as the synthesis solvent, which promotes controlled nucleation and crystal growth through its interaction with 2-methylimidazole ligands.^13,14^

**Figure 1:**
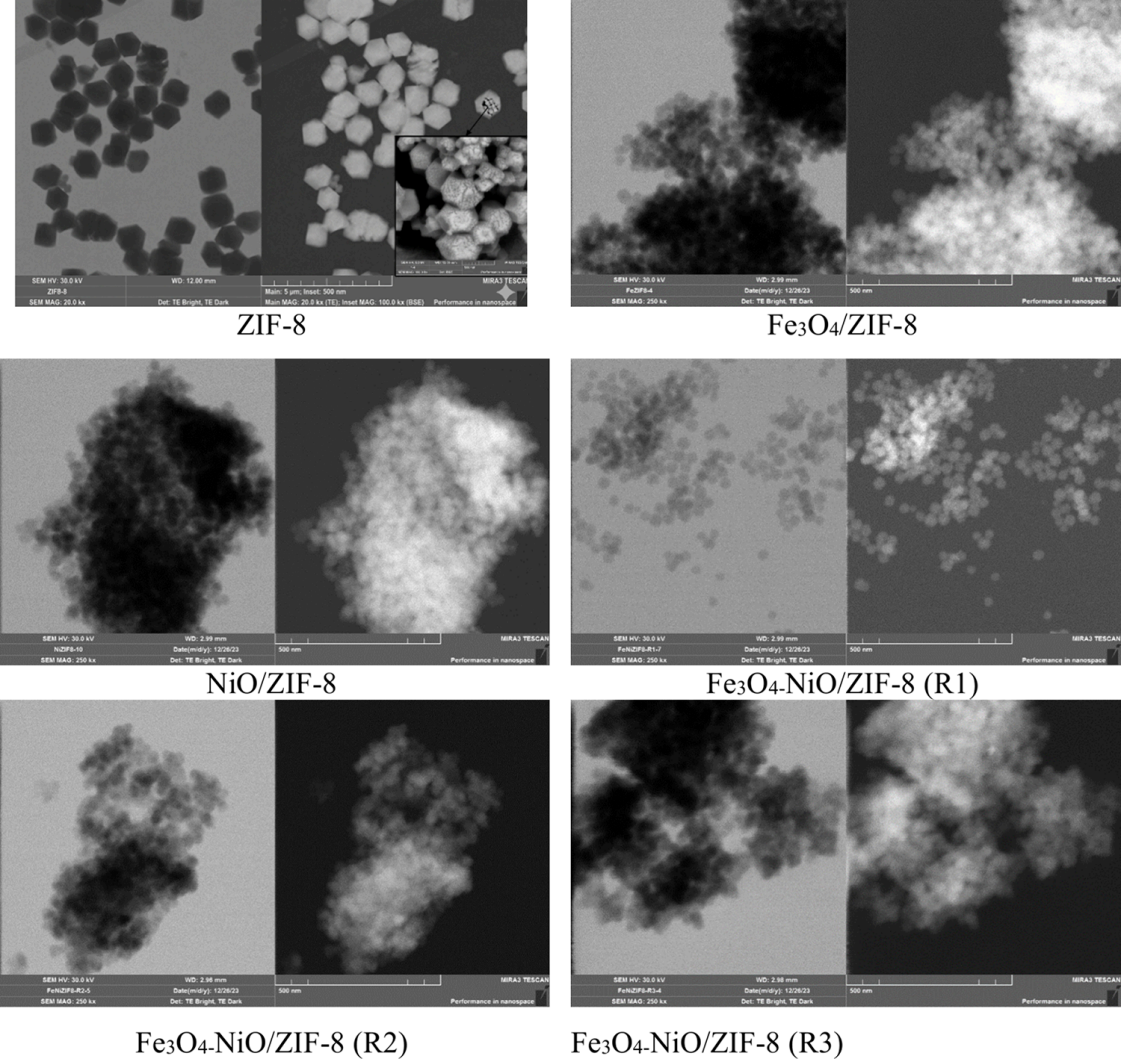
STEM images of ZIF-8 and metal oxide/ZIF-8 samples.

**Table 1:** XRF Analysis Results and Average Crystal Size of Synthesized Samples

	elemental content wt %				
samples	Zn^a^	Fe^b^	Ni^a^	molar ratio (Zn:Fe:Ni)	average crystal size (nm)
ZIF-8	25.43			1:0:0	43.5
Fe_3_O_4_/ZIF-8	27.05	0.90		1:0.04:0	18.2
NiO/ZIF-8	27.50		2.33	1:0:0.09	20.1
Fe_3_O_4‑_NiO/ZIF-8-R1	23.60	1.60	2.40	1:0.08:0.11	19.8
Fe_3_O_4‑_NiO/ZIF-8-R2	24.91	1.40	0.44	1:0.06:0.17	18.3
Fe_3_O_4‑_NiO/ZIF-8-R3	24.96	0.60	1.63	1:0.03:0.07	18.7

a Composition determined by ICP-MS.

b Composition determined by XRF.

Following the incorporation of Fe_3_O_4_ and NiO, the characteristic polyhedral morphology of ZIF-8 is largely preserved across all samples, indicating that the framework remains structurally stable under the applied synthesis conditions. Similar observations have been reported for metal-oxide-modified ZIF-8 systems, where the host structure is retained despite guest incorporation.^15^ Nevertheless, slight variations in particle distribution and morphology are observed, particularly in the bimetallic samples (R1–R3), where the Fe/Ni ratio was systematically adjusted.

The morphological characteristics observed in the STEM images are in good agreement with the elemental compositions determined by ICP-MS and XRF analyses (Table 1). The pristine ZIF-8 sample exhibited a Zn content of 25.43 wt %, which is close to the theoretical value (28.73 wt %) expected for ZIF-8, confirming the successful synthesis of the parent framework. Following metal incorporation, variations in Zn content and metal loading were observed, indicating the successful introduction of Fe and/or Ni species into the ZIF-8 structure.

For the monometallic composites, Fe_3_O_4_/ZIF-8 contained 0.90 wt % Fe and NiO/ZIF-8 contained 2.33 wt % Ni, confirming effective loading of the respective metal oxides. The incorporation of these species was accompanied by a significant reduction in average crystal size from 43.5 nm for pristine ZIF-8 to 18.2 and 20.1 nm for Fe_3_O_4_/ZIF-8 and NiO/ZIF-8, respectively. This reduction suggests that the presence of metal species influences the nucleation and growth behavior of ZIF-8 crystals during synthesis.

Among the bimetallic composites, Fe_3_O_4_·NiO/ZIF-8-R1 exhibited the highest metal loading, with 1.60 wt % Fe and 2.40 wt % Ni, corresponding to a molar ratio of Zn:Fe:Ni = 1:0.08:0.11. This sample displayed a relatively homogeneous morphology and an average crystal size of 19.8 nm, suggesting a uniform distribution of both metal species throughout the framework. In contrast, Fe_3_O_4_·NiO/ZIF-8-R2, despite containing a comparable Fe content (1.40 wt %), showed a considerably lower Ni content (0.44 wt %) and a slightly reduced Zn content (24.91 wt %). The observed morphological irregularities and crystal size of 18.3 nm may indicate stronger interactions between Fe species and the ZIF-8 framework, potentially leading to local structural distortions or partial replacement of Zn sites.^16^ Fe_3_O_4_·NiO/ZIF-8-R3 exhibited intermediate elemental composition values (0.60 wt % Fe and 1.63 wt % Ni) and an average crystal size of 18.7 nm, resulting in morphological features that were intermediate between those of R1 and R2.

The calculated Zn:Fe:Ni molar ratios further demonstrate that the relative amounts of Fe and Ni incorporated into the framework vary significantly among the composites. These differences are reflected in the crystal size and particle morphology, indicating that the Fe/Ni ratio influences both metal dispersion and crystal growth behavior.

Overall, the combined STEM, ICP-MS, and XRF results confirm the successful incorporation of Fe_3_O_4_ and NiO species into the ZIF-8 framework while preserving the characteristic crystalline structure. Furthermore, the metal composition and Fe/Ni molar ratio play a crucial role in determining crystal size, metal distribution, and the extent of structural modification within the resulting composites.

### 3.2 X-ray Diffraction Analysis of ZIF-8 and Its Composites

Figure 2 presents the XRD profiles of all synthesized materials. The diffraction profile presented in Figure 2a reveals that pristine ZIF-8 exhibits several sharp peaks at low diffraction angles, which are assignable to the (011), (112), (022), and (222) crystallographic planes. These reflections are consistent with the characteristic crystallinity of the ZIF-8 framework, confirming its successful formation.^15^ The diffraction patterns of Fe_3_O_4_/ZIF-8, NiO/ZIF-8, and their composites (R1, R2, R3), shown in Figure 2b–d, also retain the major peaks attributed to ZIF-8. The retention of these signals confirms that the parent structural network remains intact, even after metal oxide incorporation. This is further supported by the STEM images, which show that particle morphology remains largely unchanged after metal addition.

**Figure 2:**
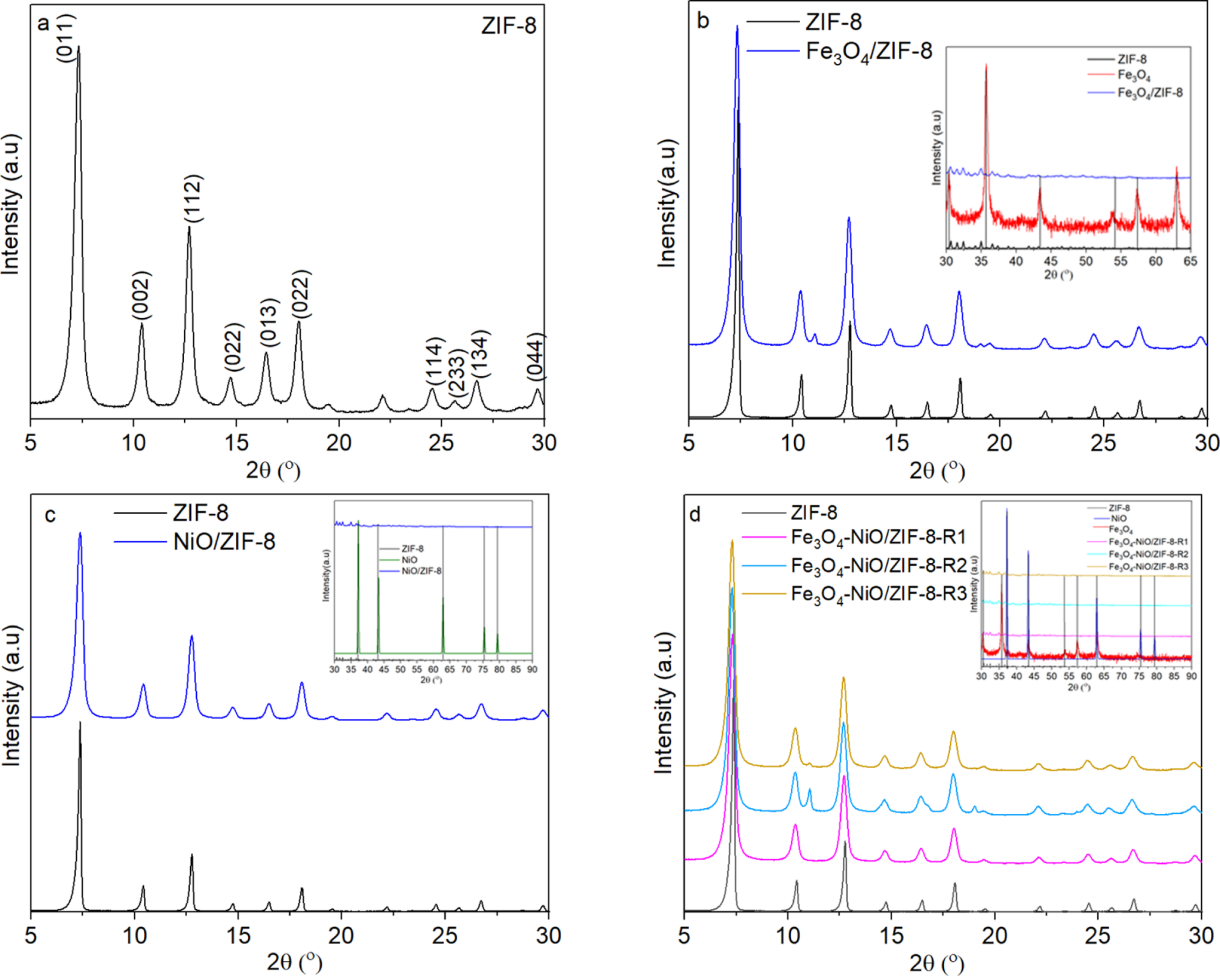
XRD patterns of (a) ZIF-8; (b) ZIF-8, Fe_3_O_4_, and Fe_3_O_4_/ZIF-8; (c) ZIF-8, NiO, and NiO/ZIF-8; and (d) ZIF-8, NiO, Fe_3_O_4_, R1, R2, and R3.

Figure 2b presents the XRD patterns of ZIF-8, pristine Fe_3_O_4_, and the Fe_3_O_4_/ZIF-8 composite. The pristine Fe_3_O_4_ exhibited characteristic diffraction peaks at 2θ values of 30.4°, 35.7°, 43.2°, 53.8°, 57.2°, and 62.9°, corresponding to the (220), (311), (400), (422), (511), and (440) crystal planes of Fe_3_O_4_, respectively.^16^ Following the incorporation of Fe_3_O_4_ into the ZIF-8 framework, the characteristic diffraction peaks of ZIF-8 remained largely preserved, indicating that the crystalline structure of ZIF-8 was maintained after composite formation. In addition, only weak Fe_3_O_4_ reflections were observed in the XRD pattern of Fe_3_O_4_/ZIF-8. The low intensity of these peaks can be attributed to the relatively low Fe_3_O_4_ loading in the composite, as confirmed by the metal content analysis presented in Table 1.

Similarly, Figure 2c compares the XRD patterns of ZIF-8, pristine NiO, and the NiO/ZIF-8 composite. Pristine NiO displayed characteristic diffraction peaks at 2θ values of 37.2°, 43.2°, 62.9°, 75.4°, and 79.4°, which are indexed to the (111), (200), (220), (311), and (222) crystal planes of NiO, respectively.^17^ These characteristic NiO reflections were also detected in the NiO/ZIF-8 composite, together with the diffraction peaks of ZIF-8. However, the NiO peaks appeared with significantly lower intensity, likely due to the low amount and high dispersion of NiO particles within the ZIF-8 matrix.

The XRD patterns of the Fe_3_O_4_–NiO/ZIF-8 composites with different metal oxide loadings (R1, R2, and R3) are presented in Figure 2d. The diffraction peak characteristics of the ZIF-8 framework are clearly observed in all composite samples, indicating that the crystalline structure of ZIF-8 was preserved after the incorporation of Fe_3_O_4_ and NiO nanoparticles. The persistence of these reflections demonstrates the high structural stability of the ZIF-8 framework during the synthesis process.

In addition to the ZIF-8 reflections, weak diffraction peaks corresponding to Fe_3_O_4_ and NiO phases can be identified in the enlarged XRD patterns shown in the inset of Figure 2d. The relatively low intensity of Fe_3_O_4_ and NiO reflections is attributed to their low loading amounts in the composites, consistent with the metal content results presented in Table 1.

Among the composite samples, R1, R2, and R3 exhibit similar diffraction profiles with no noticeable shift in the characteristic ZIF-8 peaks after metal incorporation. The absence of significant peak displacement suggests that the incorporation of Fe_3_O_4_ and NiO does not alter the crystal lattice of ZIF-8, indicating that the metal oxides are primarily dispersed on or within the porous framework rather than substituting into the ZIF-8 crystal structure.

Moreover, slight peak broadening is observed in the composite samples compared with pristine ZIF-8, which may be associated with reduced crystallite size and the introduction of microstrain or minor structural disorder during the incorporation of metal oxide nanoparticles. Overall, the XRD results confirm the successful formation of Fe_3_O_4_–NiO/ZIF-8 composites while maintaining the structural integrity of the parent ZIF-8 framework.

The crystallite size data (Table 1) further clarify the influence of metal incorporation on crystal development. Pristine ZIF-8 exhibits a relatively large crystallite size (43.5 nm), indicative of unrestricted crystal growth under the selected synthesis conditions. In contrast, all metal-modified samples display significantly smaller sizes in the range of 18.2–20.1 nm. This reduction suggests that the introduction of Fe_3_O_4_ and NiO alters the crystallization pathway by affecting nucleation and growth kinetics, likely through additional nucleation sites or framework–metal interactions. The relatively narrow size distribution among the bimetallic samples (R1–R3) indicates that variations in the Fe/Ni ratio have a limited effect on crystallite size compared to the overall impact of metal incorporation. Similar trends have been widely reported in the literature.^15,16^

Overall, the combined STEM, ICP-MS, and XRF results confirm that metal oxide incorporation is successfully achieved without compromising the ZIF-8 framework. At the same time, the Fe/Ni ratio plays a critical role in governing metal dispersion and inducing subtle structural variations within the composites.

### 3.3 FTIR Spectroscopic Analysis of the Samples

The FTIR spectra of ZIF-8, Fe_3_O_4_/ZIF-8, and NiO/ZIF-8 are presented in Figure 3a,b, providing complementary structural insights alongside the XRD, ICP-MS, and XRF results. The spectra in Figure 3a confirm that all samples retain the characteristic vibrational features of the ZIF-8 framework, indicating that the incorporation of Fe_3_O_4_ and NiO does not disrupt the imidazolate structure.

**Figure 3:**
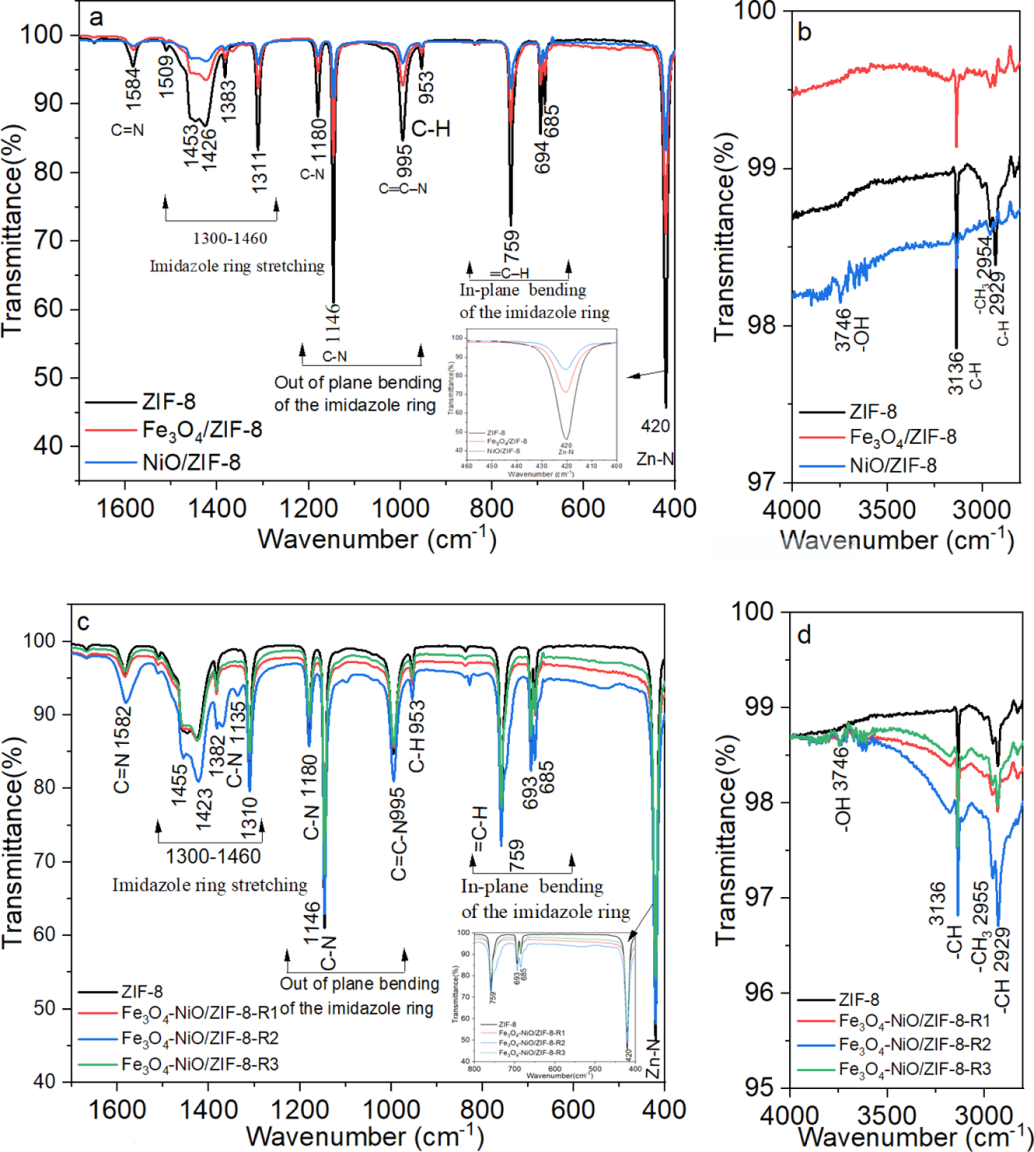
(a) FTIR spectra of ZIF-8, NiO/ZIF-8, and Fe_3_O_4_/ZIF-8 samples in the 400–1700 cm^–1^ region; (b) FTIR spectra of ZIF-8, NiO/ZIF-8, and Fe_3_O_4_/ZIF-8 samples in the 2800–4000 cm^–1^ region; (c) FTIR spectra of ZIF-8 and Fe_3_O_4_–NiO/ZIF-8­(R1–R3) samples in the 400–1700 cm^–1^ region; and (d) FTIR spectra of ZIF-8 and Fe_3_O_4_–NiO/ZIF-8 (R1–R3) samples in the 2800–4000 cm^–1^ region.

A distinct absorption band at approximately 420 cm^–1^ is assigned to the Zn–N stretching vibration, confirming the preservation of the coordination environment of ZIF-8 in the composite structures.^18,19^ The peak at 1584 cm^–1^ corresponds to the C=N stretching vibration of the imidazolate linker.^20,21^ This band appears sharp in pristine ZIF-8 but shows a noticeable decrease in intensity after the incorporation of Fe_3_O_4_ and NiO, suggesting interactions between the metal oxides and the framework.

The band located at 1146 cm^–1^, attributed to C–N stretching vibrations,^22,23^ also decreases in intensity upon composite formation. Additional peaks at 1147 and 1180 cm^–1^ are assigned to in-plane C–N stretching modes.^24^ The bands observed at 1311 and 953 cm^–1^ correspond to C–H bending vibrations of the imidazole ring,^24^ while the peak around 995 cm^–1^ is associated with C=C–N twisting vibrations.^25^ The absorption band at approximately 753 cm^–1^ is assigned to =C–H bending,^25^ whereas the bands at 685–694 cm^–1^ are attributed to the puckering of the imidazole ring.

Furthermore, the in-plane and out-of-plane bending vibrations of the imidazole ring are observed in the regions of 694–756 and 953–1148 cm^–1^, respectively,^26^ as clearly indicated in Figure 3a. The absorption bands at 2929 and 3136 cm^–1^ (Figure 3b) are attributed to aliphatic and aromatic C–H stretching vibrations of the imidazole ring, respectively.^27^ A high-wavenumber band centered at approximately 3746 cm^–1^ is assigned to O–H stretching vibrations, indicating the presence of hydroxyl groups and hydrogen bonding interactions.^28^

Figure 3a also shows that the overall intensity of the ZIF-8 characteristic peaks decreases after the incorporation of Fe_3_O_4_ and NiO (5 mol %). This decrease is more pronounced for the NiO/ZIF-8 sample compared to Fe_3_O_4_/ZIF-8, suggesting a stronger interaction or higher incorporation degree of NiO within the ZIF-8 framework, which is consistent with the compositional analysis presented in Table 1.

The FTIR spectra of bare Fe_3_O_4_ and NiO nanoparticles are given in Figure S1a–c. As shown in Figure S1a–b, Fe_3_O_4_ exhibits multiple characteristic bands in the low-wavenumber region (approximately 400–600 cm^–1^), associated with Fe–O vibrations. In contrast, Figure S1c shows that NiO presents a main absorption band around 405 cm^–1^ along with a broad feature in the range of 975–1165 cm^–1^. These features confirm the successful formation of the respective metal oxides prior to their incorporation into the ZIF-8 structure.

Figure 3c,d provides a detailed representation of the fingerprint and high-wavenumber regions of the FTIR spectra for Fe_3_O_4_–NiO/ZIF-8 samples. Figure 3c shows that the characteristic vibrational bands of the imidazolate framework, particularly within the 1300–1460 cm^–1^ region, are preserved in all samples, confirming that the fundamental ZIF-8 structure remains intact. However, noticeable reductions in band intensity and slight broadening effects are evident in the composite samples, indicating interactions between the framework and the incorporated metal oxides.

These spectral changes correlate well with the compositional data presented in Table 1. In particular, the Fe_3_O_4_–NiO/ZIF-8 samples exhibit varying Zn:Fe:Ni molar ratios (1:0.03:0.07 to 1:0.08:0.11), which influence the degree of perturbation in the vibrational features. The sample with higher Ni content (Fe_3_O_4_–NiO/ZIF-8-R1) shows more pronounced attenuation in the C=N (∼1582 cm^–1^) and C–N (∼1146 cm^–1^) bands, suggesting stronger interactions between NiO and the ZIF-8 framework. This observation is consistent with the earlier discussion, where NiO incorporation led to greater spectral intensity loss compared to Fe_3_O_4_ alone.

Additionally, the peaks associated with C=C–N vibrations (∼995 cm^–1^) and imidazole ring bending modes (600–800 cm^–1^ region) exhibit subtle distortions and decreased intensity in the composite samples. These effects can be attributed to changes in the local electronic environment and partial restriction of ring dynamics upon metal oxide incorporation. Despite these modifications, the persistence of all ZIF-8 framework-related bands confirms that the ZIF-8 topology is preserved.

Figure 3d further highlights the high-wavenumber region, where O–H and C–H stretching vibrations are located. The broad O–H band (∼3746 cm^–1^) becomes slightly more prominent in the composites, indicating increased surface hydroxyl groups or adsorbed moisture, likely associated with the presence of Fe_3_O_4_ and NiO. Meanwhile, the aromatic (∼3136 cm^–1^) and aliphatic (∼2929–2955 cm^–1^) C–H stretching bands show minor intensity decreases, supporting the presence of interfacial interactions.

Furthermore, the crystallite size data in Table 1 provide additional insight into these observations. The pristine ZIF-8 sample exhibits a larger average crystal size (43.5 nm), whereas the composite samples show significantly reduced sizes (18.2–20.1 nm). This reduction suggests that metal oxide incorporation affects crystal growth, leading to smaller particles with higher surface area. The increased surface-to-volume ratio in these smaller crystallites likely enhances interfacial contact between ZIF-8 and the metal oxides, thereby contributing to the observed attenuation and broadening of FTIR bands.

Overall, the combined FTIR and compositional analysis demonstrates that although the incorporation of Fe_3_O_4_ and NiO does not disrupt the ZIF-8 framework, it induces measurable changes in vibrational intensity, band shape, and local chemical environment. Among the studied samples, NiO-rich compositions exhibit a more pronounced effect, consistent with their higher incorporation level and stronger interaction with the framework.

### 3.4 XPS Analysis of the Samples

The XPS survey spectrum of the Fe_3_O_4_/ZIF-8 composite is presented in Figure 4a. The survey results confirm the presence of Zn 2p_3/__2_ (9.47%), C 1s (61.52%), N 1s (26.04%), and O 1s (2.96%) elements in the sample, in line with the predicted composition of the Fe_3_O_4_/ZIF-8 hybrid material.

**Figure 4:**
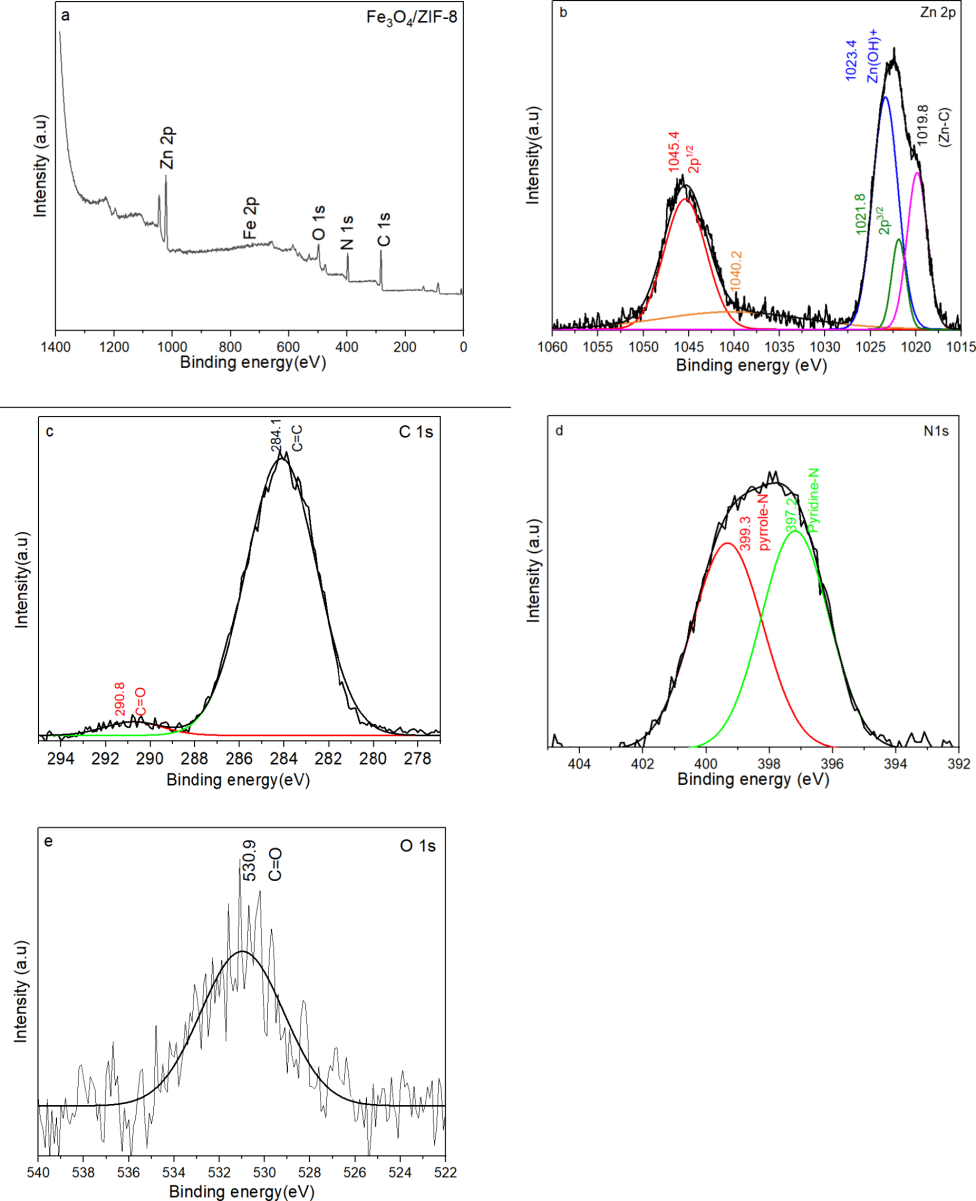
X-ray photoelectron spectroscopy characterization of the Fe_3_O_4_/ZIF-8 sample showing (a) full survey spectrum and deconvoluted core levels of Zn 2p (b), C 1s (c), N 1s (d), and O 1s (e).

The high-resolution Zn 2p is presented in Figure 4b. The deconvoluted peaks appear at binding energies of 1045.4, 1040.2, 1023.4, 1021.8, and 1019.8 eV. The two main peaks located at 1021.8 and 1045.4 eV are assigned to Zn 2p_3/2_ and Zn 2p_1/2_, respectively. The energy separation between these peaks is 23.6 eV, which is characteristic of Zn^2+^ species,^29^ confirming the predominant oxidation state of zinc in the sample.

In addition to these primary features, the Zn 2p spectrum exhibits a component at a higher binding energy of 1023.4 eV, which is attributed to Zn–OH species,^30^ indicating the presence of surface hydroxyl groups on the material. This assignment is consistent with the FTIR results, where a broad absorption band observed in the 3200–3600 cm^–1^ region further supports the presence of hydroxyl functionalities associated with adsorbed water or surface −OH groups.

Therefore, the combined FTIR and XPS analyses confirm the formation of Zn–OH species on the surface of the ZIF-8-based structure. A lower binding energy component at 1019.5 eV is assigned to Zn–C bonding,^31^ suggesting an interaction between zinc centers and the carbon-based framework. Furthermore, the broad peak centered at approximately 1040.2 eV is associated with Zn^2+^ satellite features,^32^ reflecting the complex electronic structure of zinc in the material.

Deconvolution of the C 1s signal (Figure 4c) reveals two dominant contributions centered at 284.1 and 290.8 eV, which are associated with C=C and C=O bonding environments, respectively.^33,34^ The N 1s profile (Figure 4d) revealed two types of nitrogen species, including pyridine-N (397.2 eV) and pyrrole-N (399.3 eV).^35^ In the O 1s spectrum (Figure 4e), a well-defined component located at 530.9 eV is observed and attributed to oxygen species in C=O functional groups.^36^

Additionally, although the Fe 2p XPS signal is relatively weak due to the low Fe content, no distinct peaks could be detected, as shown in Figure S2a. These findings confirm the presence of the characteristic functional groups of ZIF-8; however, the incorporation of Fe_3_O_4_ nanoparticles into the ZIF-8 framework could not be verified from Figure S2a.

The XPS analysis results of the NiO/ZIF-8 sample are presented in Figure 5a. The surface elemental composition of NiO/ZIF-8 consists of 0.47% O 1s, 60.39% C 1s, 27.47% N 1s, 11.40% Zn 2p_3/2_, and 0.26% Ni 2p_3/2_.

**Figure 5:**
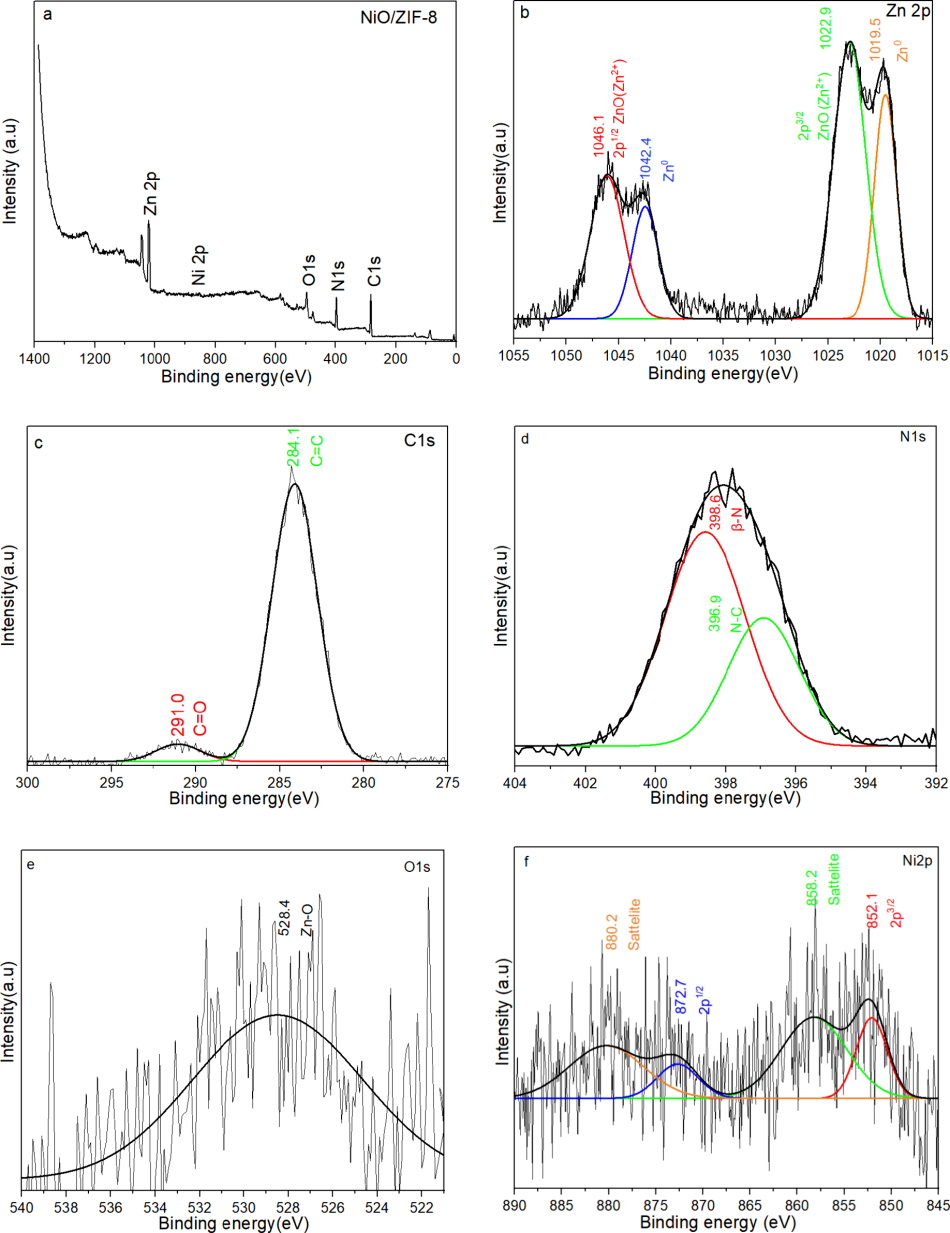
X-ray photoelectron spectroscopy characterization of the NiO/ZIF-8 sample showing (a) full survey spectrum and deconvoluted core levels of Zn 2p (b), C 1s (c), N 1s (d), O 1s (e) and Ni 2p (f).

Figure 5b illustrates the high-resolution XPS spectrum of Zn 2p. The spectrum exhibits two primary doublets centered at approximately 1021 and 1044 eV, corresponding to the spin–orbit splitting of Zn 2p_3/2_ and Zn 2p_1/2_, respectively. A rigorous peak-fitting analysis identifies two distinct chemical environments for Zn. The most prominent peaks, centered at 1022.9 eV (Zn 2p_3/2_) and 1046.1 eV (Zn 2p_1/2_), are ascribed to the divalent oxidation state (Zn^2+^).^37^ This finding confirms that the zinc ions are successfully integrated into the zeolitic imidazolate framework, maintaining their tetrahedral coordination with the organic linkers even after the incorporation of the NiO phase. The observed binding energy is consistent with the established parameters for Zn–N coordination in ZIF-8 materials.

Intriguingly, the spectrum reveals a secondary electronic state at 1019.5 and 1042.4 eV, which corresponds to metallic zinc (Zn^0^).^38–40^ In the context of a NiO/ZIF-8 composite, the emergence of a Zn^0^ component is a significant indicator of interfacial phenomena. This can be interpreted as a consequence of localized electronic density redistribution at the junction between the NiO nanoparticles and the ZIF-8 support. Such a shift toward lower binding energies often implies an electron-rich environment, potentially arising from a partial reduction process during the hybrid’s synthesis or as a result of synergistic charge transfer between the metal oxide and the MOF. The slight variation in the Zn^2+^ binding energy relative to pristine ZIF-8 further substantiates the presence of a strong electronic coupling within the NiO/ZIF-8 heterojunction, which is critical for enhancing the material’s functional performance in target applications.

In the C 1s region (Figure 5c), two dominant contributions are identified at binding energies of 284.1 and 291.0 eV, which are assigned to C=C and C=O functional groups, respectively.^33,34^ The N 1s profile (Figure 5d) consists of two well-resolved components centered at 398.6 eV^41^ and 396.9 eV,^42^ attributable to β-N and N–C chemical environments derived from the organic linker. Notably, the signal at 398.6 eV is characteristic of β-N species and is commonly associated with O–Ni–N coordination within the composite structure.^43^ The O 1s (Figure 5e) shows a prominent peak at 528.48 eV, attributed to Zn–O and/or Ni–O bonds within the composite. Although the Ni 2p signal was noisy, the Ni 2p region (Figure 5f) comprises four peaks: the Ni 2p_3/2_ main peak and its satellite at 852.1 and 858.2 eV, and the Ni 2p_1/2_ main peak and its satellite at 872.1 and 880.1 eV, respectively, confirming the presence of Ni^2+^ ions in the NiO/ZIF-8 sample.^44^

The XPS analysis results of the Fe_3_O_4_–NiO/ZIF-8 (R1) composite are illustrated in Figure 6. The survey spectrum (Figure 6a) shows that the sample contains C 1s (62.11%), N 1s (25.43%), O 1s (2.40%), Zn 2p_3/2_ (9.79%), and Ni 2p_3/2_ (0.24%) elements. The Fe signal was not detected, which can be attributed to its relatively low concentration within the ZIF-8 structure, as seen in Table 1. The Zn 2p spectrum of Fe_3_O_4_–NiO/ZIF-8 (Figure 6b) indicates two peaks at 1044.2 and 1020.9 eV, corresponding to Zn 2p_1/2_ and Zn 2p_3/2_, respectively, relating to the presence of divalent zinc species (Zn^2+^) within the Fe_3_O_4_–NiO/ZIF-8 composite.^45^

**Figure 6:**
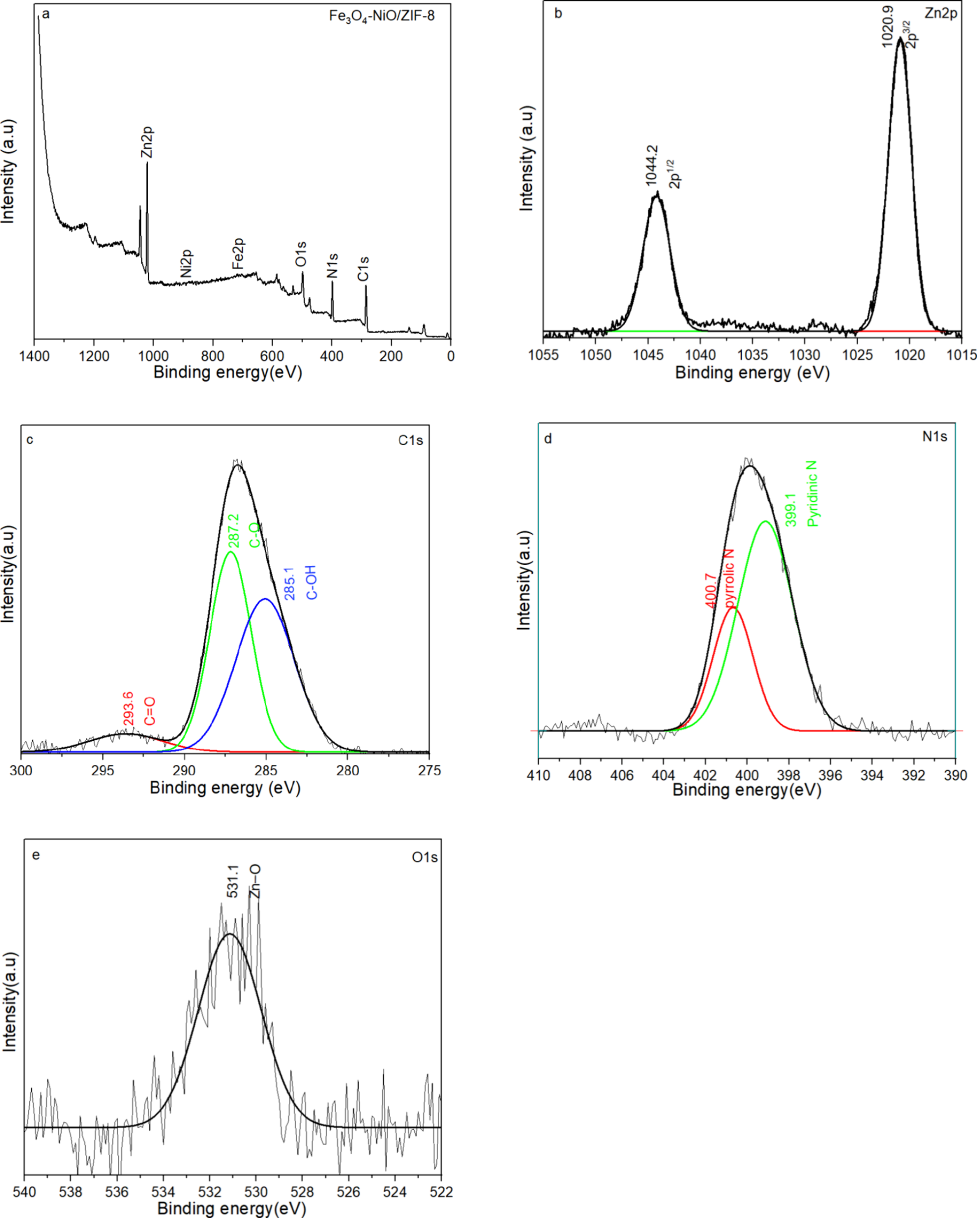
X-ray photoelectron spectroscopy characterization of the Fe_3_O_4_–NiO/ZIF-8 (R1) sample showing (a) full survey spectrum and deconvoluted core levels of (b) Zn 2p, (c) C 1s, (d) N 1s, and (e) O 1s.

The high-resolution C 1s XPS profile of the Fe_3_O_4_–NiO/ZIF-8 composite (Figure 6c) can be deconvoluted into three components centered at 285.1, 287.2, and 293.6 eV, which are attributed to C–OH, C–O, and C=O functional groups, respectively.^46,47^ The corresponding N 1s spectrum (Figure 6d) contains two well-defined contributions at 399.1 and 400.7 eV, assigned to pyridinic and pyrrolic nitrogen species originating from the organic linker of the ZIF-8 framework.^48^ In the O 1s region (Figure 6e), a prominent feature located at 531.1 eV is observed and is indicative of lattice oxygen associated with Zn–O, Ni–O, and Fe–O bonding environments within the hybrid material. In the Fe 2p region (Figure S2c), very weak and broad features are observed at approximately 705.6 and 717.9 eV, corresponding to Fe 2p_3/2_ and Fe 2p_1/2_,^49^ along with a satellite feature around 726.1 eV. However, due to the low Fe content and poor signal-to-noise ratio, these peaks are not well-resolved. Therefore, while the presence of Fe species is suggested, the successful incorporation of Fe_3_O_4_ nanoparticles into the ZIF-8 framework cannot be conclusively confirmed from the XPS data alone.

### 3.5 Raman Spectroscopic Analysis of the Samples

Raman spectral profile of ZIF-8, in which characteristic vibrational modes arising from the imidazolate linker and its methyl groups are clearly observed. A detailed description of the Raman band assignments obtained from Figure S3a is provided in Table S1. Intense signals located at 74, 168, 686, 1146, and 1458 cm^–1^ are attributed to Zn–N stretching, deformation of the imidazole ring, C5–N stretching vibrations, and methyl group bending modes, respectively. These spectral features are consistent with those reported by Kumari et al., and, when considered alongside the XRD, FTIR, and XPS results, collectively confirm the successful construction of the ZIF-8 framework.^50^

In the Raman spectrum of the Fe_3_O_4_/ZIF-8 composite (Figure S3b), additional peaks are observed at 300, 532, and 661 cm^–1^. These peaks correspond to the E_g_, T_2g_(2), and A_1g_ vibrational modes of magnetite, respectively, and are characteristic of Fe–O vibrations in Fe_3_O_4_ nanoparticles.^51^ These signals, alongside the preserved ZIF-8 bands, indicate that Fe_3_O_4_ particles are successfully incorporated into the ZIF-8 structure, corroborating the findings from XRD analysis.

The Raman spectra of NiO/ZIF-8 (Figure S3c) display the characteristic bands of ZIF-8, along with additional peaks at approximately 400, 530, 730, 900, and 1090 cm^–1^, which are attributed to Ni–O vibrations in NiO.^52^ These findings, consistent with the XRD results, confirm the successful incorporation of NiO particles into the ZIF-8 framework.

Raman spectra of ZIF-8 composites containing different proportions of Fe_3_O_4_ and NiO are shown in Figure 7. While the main ZIF-8 bands remain largely unchanged, the presence of distinct NiO and Fe_3_O_4_ peaks further confirms that both metal oxides are effectively integrated into the structure, with their relative intensities reflecting the varying loading levels.

**Figure 7:**
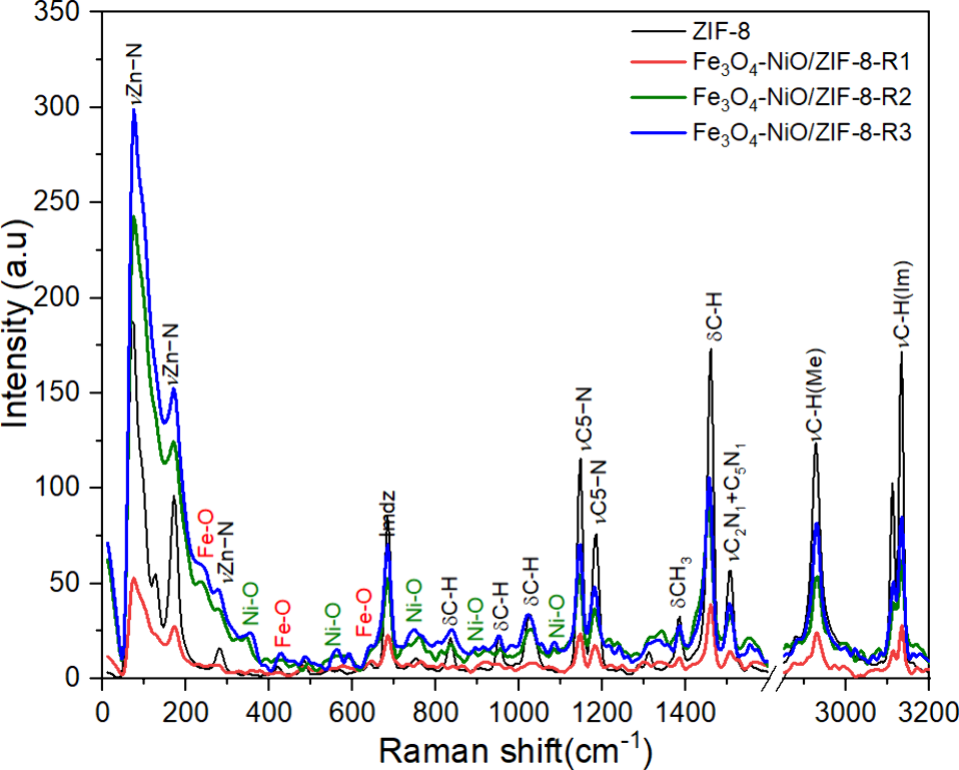
Raman characterization of ZIF-8 before and after Fe_3_O_4_–NiO incorporation.

### 3.6 Textural Properties and Porosity Analysis by N_2_ Adsorption–Desorption

To evaluate the porous architecture of the synthesized materials, nitrogen (N_2_) adsorption–desorption measurements were conducted at 77 K. The resulting isotherms and corresponding pore size distributions are illustrated in Figure 8a,b, respectively, while key textural parameters are compiled in Table 2.

**Figure 8:**
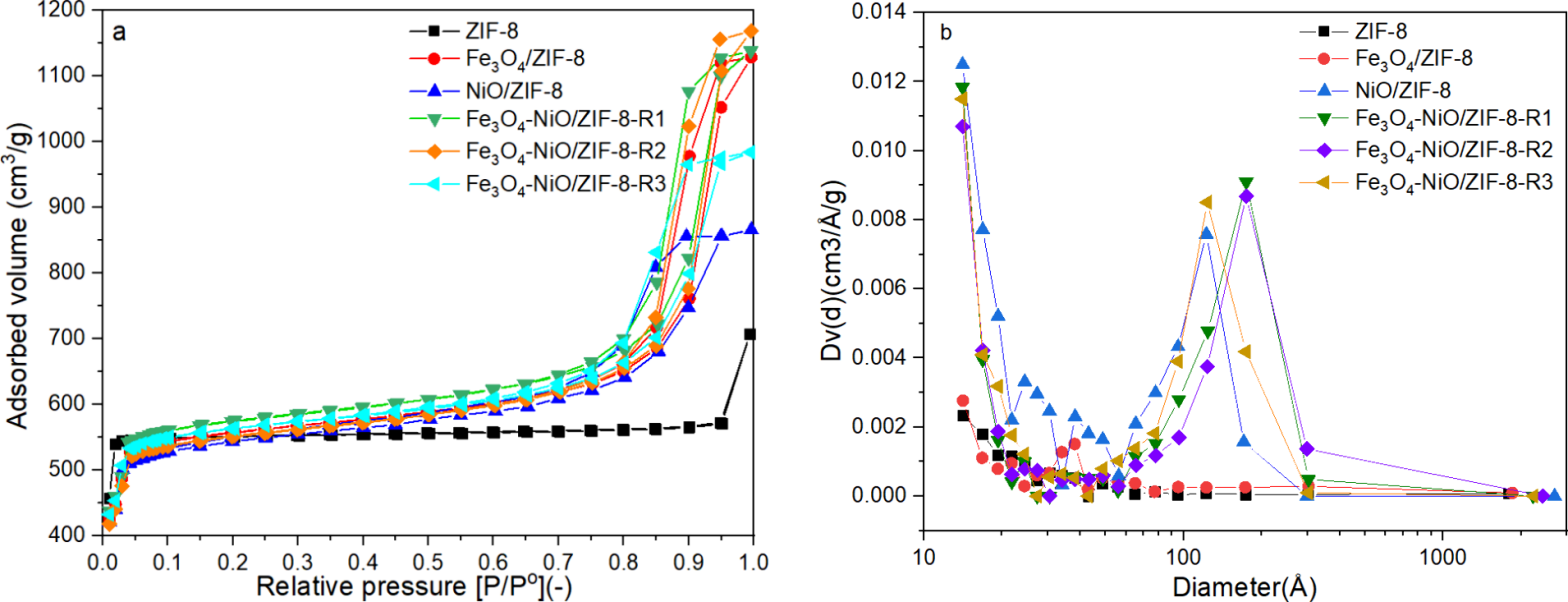
(a) N_2_ adsorption–desorption isotherms obtained by volumetric analysis. (b) Pore size distribution derived from desorption.

**Table 2:** Surface Area and Porosity Characteristics Derived from N_2_ Sorption Measurements

sample	*S*_BET_ ^a^(m^2^/g)	*V*_Total_ ^b^(cm^3^/g)	V_Micro_ ^c^(cm^3^/g)	*D* ^d^(Å)
ZIF-8	2088	1.096	0.840	21.0
Fe_3_O_4_/ZIF-8	563	0.629	0.228	44.7
NiO/ZIF-8	2016	1.343	0.724	26.6
Fe_3_O_4_–NiO/ZIF-8-R1	2016	1.765	0.799	35.0
Fe_3_O_4_–NiO/ZIF-8-R2	2046	1.812	0.755	35.4
Fe_3_O_4_–NiO/ZIF-8-R2	2111	1.526	0.785	28.9

a Surface area calculated using the multipoint BET method.

b Total pore volume determined at P/Po = 0.99.

c Micropore volume determined by the BJH method.

d Average pore diameter determined by the DR method.

The unmodified ZIF-8 framework demonstrates a remarkably high BET surface area (*S*_BET_ = 2088 m^2^ g^–1^), accompanied by a total pore volume (*V*_Total_) of 1.096 cm^3^ g^–1^ and a dominant microporous contribution (*V*_Micro_ = 0.84 cm^3^ g^–1^). The average pore diameter of 21.0 Å is consistent with reported values for ZIF-8, confirming the integrity of its sodalite-type microporous network prior to composite formation. All synthesized samples display type IV isotherms according to IUPAC classification,^53^ each featuring a pronounced H3-type hysteresis loop within the P/P^0^ range of 0.7–1.0. This hysteresis behavior indicates capillary condensation occurring within mesoporous domains, which coexist with the intrinsic microporous structure of the ZIF-8 framework. Consequently, a hierarchical dual-porosity system is formed, which is attributed to interparticle void spaces and textural reorganization induced by metal oxide incorporation, thereby facilitating enhanced mass transport.

Importantly, the observed deviation from the typical type I isotherm of pristine ZIF-8 is not related to structural collapse of the framework. This is supported by XRD results, which confirm the preservation of crystallinity, as well as by the retention of significant microporous volume across all samples. Therefore, the type IV adsorption behavior is primarily ascribed to the development of interparticle mesoporosity and textural modifications rather than degradation of the ZIF-8 framework.

Incorporation of Fe_3_O_4_ into the ZIF-8 host (Fe_3_O_4_/ZIF-8) results in a reduction of *S*_BET_ to 563 m^2^ g^–1^, with a micropore volume of 0.228 cm^3^ g^–1^ and a substantially increased mean pore diameter of 44.7 Å. Despite this reduction, the retention of appreciable microporosity suggests that Fe_3_O_4_ nanoparticles are largely deposited at external surfaces or within intercrystalline spaces, rather than penetrating and obstructing the internal microporous channels of ZIF-8. The NiO/ZIF-8 composite, by contrast, retains a high surface area (2016 m^2^ g^–1^) with a micropore volume of 0.724 cm^3^ g^–1^ and a pore diameter of 26.6 Å, suggesting a more uniform distribution of NiO species with limited disruption to the framework’s intrinsic porosity.

For the ternary Fe_3_O_4_–NiO/ZIF-8 composites, a progressive enhancement in textural properties is observed across the R1–R3 series (Table 2). Specifically, *S*_BET_ increases from 2016 m^2^ g^–1^ (R1) to 2046 m^2^ g^–1^ (R2) and reaches 2111 m^2^ g^–1^ (R3), while total pore volumes range from 1.765 to 1.526 cm^3^ g^–1^. This trend suggests that fine-tuning the Fe_3_O_4_/NiO molar ratio governs the spatial arrangement of metal oxide phases within the hybrid framework. At higher Fe_3_O_4_ content (R3), the partial recovery of surface area beyond that of pristine ZIF-8 points to the formation of additional interparticle porosity, likely resulting from nanoscale structural rearrangements at the oxide–framework interface. The concurrent increase in total pore volume across the series further implies the progressive development of a hierarchical pore network, which is anticipated to benefit both diffusional accessibility and adsorption capacity in practical applications.

As evidenced by the pore size distribution profiles presented in Figure 8b, all composite materials display a narrow size distribution, with most pores distributed below 300 Å, indicating the preservation of a hierarchical micro/mesoporous structure. The hysteresis loops discernible in the adsorption–desorption isotherms provide further evidence for the coexistence of secondary mesoporosity, which is ascribed to interparticle void spaces and localized lattice imperfections generated upon the incorporation of Fe_3_O_4_ and NiO phases into the ZIF-8 host matrix.

### 3.7 Hydrogen Storage Performance

Textural properties of the synthesized materials were first analyzed by N_2_ adsorption–desorption measurements to establish a structural basis for evaluating hydrogen storage performance. Based on these results, volumetric H_2_ adsorption experiments were conducted at 77 K and up to 0.9 bar. Three modification strategies were considered: incorporation of Fe_3_O_4_, incorporation of NiO, and their combined incorporation in Fe_3_O_4_/NiO/ZIF-8 composites, enabling evaluation of possible synergistic effects.

The main textural parameters and hydrogen storage capacities are summarized in Table 3, while the adsorption–desorption isotherms are presented in Figures 9 and S4. The pristine ZIF-8 sample exhibited the highest BET surface area (231.6 m^2^/g), pore volume (0.045 cm^3^/g), and a mean particle size of 25.9 nm, resulting in the highest H_2_ uptake of 1.140 wt %. As shown in Figures 9a and S4a, ZIF-8 consistently demonstrates superior adsorption performance across the entire pressure range, which can be attributed to its fully accessible microporous structure.

**Table 3:** Comparative Hydrogen Storage Properties of the Studied Samples

sample	*S*_BET_(m^2^/g)	pore volume (H–K method) (cm^3^/g)	average pore diameter (nm)	average particle size (nm)	weight %H_2_ maximum
ZIF-8	231.6	0.045	2.69	25.9	1.140
Fe_3_O_4_/ZIF-8	225.8	0.044	2.74	26.6	1.132
NiO/ZIF-8	219.0	0.045	2.82	27.4	1.129
R1	222.5	0.043	2.74	26.9	1.117
R2	210.1	0.041	2.70	28.56	1.038
R3	201.2	0.041	2.72	29.83	1.001

**Figure 9:**
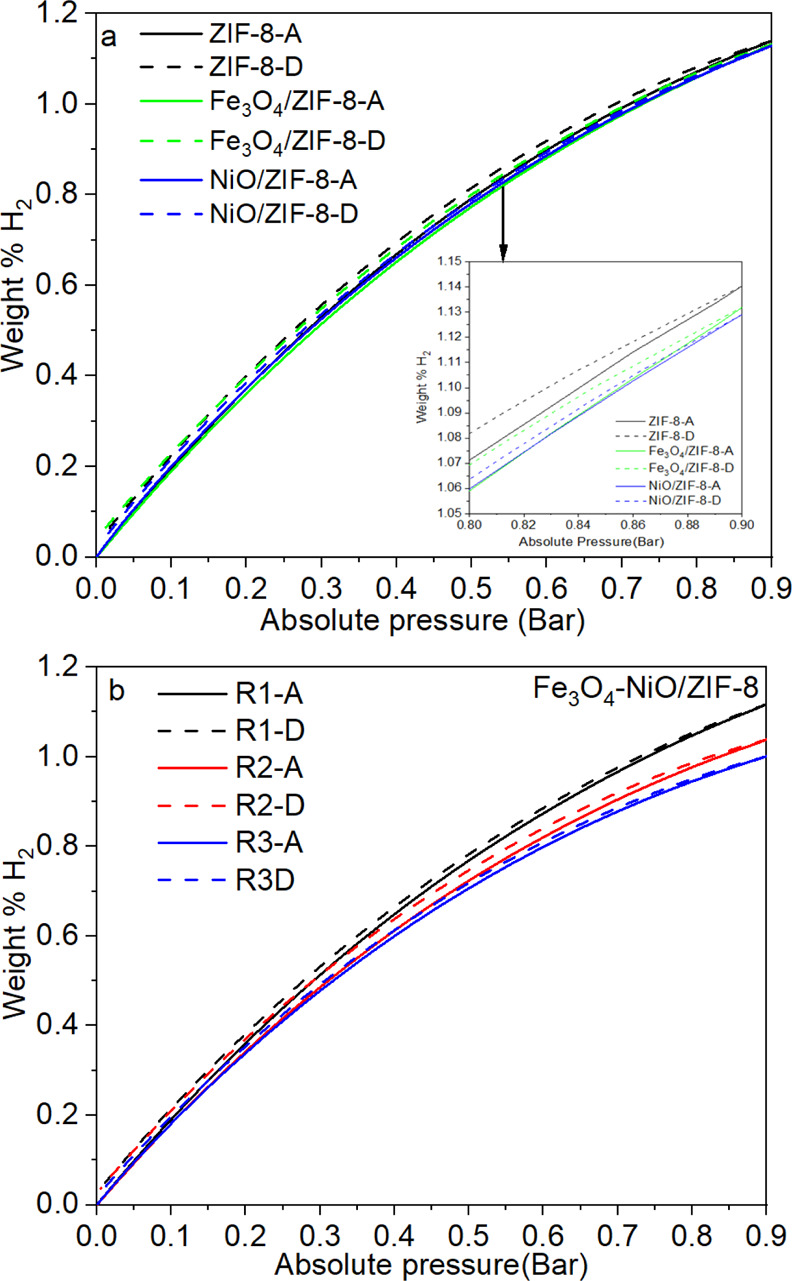
Hydrogen adsorption–desorption isotherms of (a) pristine ZIF-8, Fe_3_O_4_/ZIF-8, and NiO/ZIF-8, and (b) ternary Fe_3_O_4_–NiO/ZIF-8 composites (R1, R2, R3) measured at 77 K up to 0.9 bar. Solid and dashed lines represent adsorption (A) and desorption (D) branches, respectively. The inset in panel (a) highlights the differences in H_2_ uptake at the high-pressure region.

The introduction of metal oxide phases did not cause a significant reduction in hydrogen uptake. As observed in Figures 9a–b and S4b–d, the adsorption curves of Fe_3_O_4_/ZIF-8, NiO/ZIF-8, and R1 closely follow that of pristine ZIF-8. The maximum H_2_ uptake values were 1.132, 1.129, and 1.117 wt % for Fe_3_O_4_/ZIF-8, NiO/ZIF-8, and R1, respectively, indicating only a minor decrease. This behavior can be explained by a compensating effect: although metal oxide incorporation partially reduces surface area due to pore blockage, it also introduces additional adsorption sites that help maintain the overall storage capacity.^54^

In contrast, a more pronounced decline in H_2_ uptake is observed for the R2 and R3 samples. This trend is clearly visible in both Figures 9b and S4e–f, where the isotherms shift downward with increasing NiO content. As the Fe_3_O_4_ proportion decreases from R1 to R3, both the BET surface area and hydrogen uptake decrease systematically. The R2 sample shows a maximum uptake of 1.038 wt %, while the NiO-rich R3 sample exhibits the lowest value of 1.001 wt %. This reduction is consistent with its lower surface area (201.2 m^2^/g) and larger particle size (29.83 nm) (Table 3).

Overall, the hydrogen storage capacities follow the order: ZIF-8 (1.140 wt %) > Fe_3_O_4_/ZIF-8 (1.132 wt %) ≈ NiO/ZIF-8 (1.129 wt %) > R1 (1.117 wt %) > R2 (1.038 wt %) > R3 (1.001 wt %). The consistent trends observed in Figures 9, S4, and Table 3 indicate that the Fe_3_O_4_ phase plays a more critical role than NiO in preserving hydrogen adsorption performance.

Hydrogen adsorption in these materials is governed by both structural properties and operating conditions. At cryogenic temperatures, adsorption is mainly controlled by surface area, whereas at ambient or elevated temperatures, it also depends on the strength of interactions between hydrogen molecules and the adsorbent surface.^55–58^ In this context, the present results demonstrate that compositional modification affects performance through a balance between textural properties and surface interactions. While moderate metal oxide incorporation preserves capacity via a compensating effect, excessive NiO content leads to a dominant loss of surface area and reduced adsorption.

In line with previous studies, hydrogen storage capacities in metal–organic frameworks (MOFs) vary widely depending on pore structure, surface chemistry, and metal composition.^56,59–64^ In some systems, pore architecture governs adsorption behavior,^65–68^ whereas in others, surface properties and metal–hydrogen interactions become more significant. Additionally, temperature and pressure remain key parameters that strongly influence hydrogen storage performance.

## 4 Conclusion

ZIF-8 and its composites containing Fe_3_O_4_ and NiO were successfully synthesized via the in situ incorporation of metal oxides during the formation of the ZIF-8 framework. XRD analysis showed a slight reduction in crystallite size (18.2–20.1 nm) upon metal addition; however, the values remained comparable across all samples, indicating that Fe_3_O_4_ and NiO incorporation did not significantly alter the overall crystallinity of ZIF-8. This observation is consistent with ICP-MS and XPS results, where the binding energies of the ZIF-8 framework elements (C, N, O, Zn) remained largely unchanged, confirming the preservation of structural integrity. The oxidation states were identified as Zn^2+^, Ni^2+^, and mixed Fe^2+^/Fe^3+^, verifying the successful incorporation of metal oxides into the ZIF-8 matrix.

Textural and adsorption analyses demonstrated that hydrogen storage performance is closely related to both surface area and composition. Pristine ZIF-8 exhibited the highest hydrogen uptake (1.140 wt %) due to its fully accessible microporous structure. The incorporation of metal oxides generally preserved the adsorption capacity through a compensating effect between partial pore blockage and the creation of new adsorption sites. Among the modified samples, Fe_3_O_4_/ZIF-8 showed the best performance with a hydrogen uptake of approximately 1.13 wt %. This enhancement is attributed to the presence of Fe species, which likely strengthen interactions with hydrogen molecules. In contrast, increasing the NiO content in ternary composites (R2 and R3) led to a systematic decrease in both surface area and hydrogen uptake, highlighting the more critical role of the Fe_3_O_4_ phase in maintaining adsorption performance.

Overall, these findings demonstrate that the controlled incorporation of transition metal oxides into ZIF-8 is an effective strategy to tune its structural and hydrogen storage properties. In particular, optimizing the Fe_3_O_4_:NiO ratio is essential for achieving improved performance, making these composites promising.

## Supplementary Material



## Notes

Raman band assignments of ZIF-8, FTIR results of bare Fe_3_O_4_ and NiO nanoparticles, Raman spectra of ZIF-8 and metal oxide/ZIF-8 composites, and hydrogen adsorption–desorption isotherms for all samples (PDF)
