# Supplementary material for: Influence of Metal Oxide Incorporation on the Structure, Surface Features, and Hydrogen Storage Behavior of ZIF‑8
Source: ACS Omega. 2026 Jul 4;11(28):41732–46. doi: 10.1021/acsomega.6c01498 (PMC13392883; doi:10.1021/acsomega.6c01498)
Supplement: Supplementary file 1 [file ao6c01498_si_001.pdf]

Influence of Metal Oxide Incorporation on the Structure, Surface Features, and Hydrogen Storage Behavior of ZIF-8

Ayten Ateş[a*], Beyza Evgin[a] and Fulya Karaoğlu[a],

[a]Sivas Cumhuriyet University, Faculty of Engineering, Department of Chemical Engineering, Sivas, Turkey

*Corresponding author current address: Sivas Cumhuriyet University, Engineering Faculty, Department of Chemical Engineering, 58140 Sivas, Turkey. E-mail: ates@cumhuriyet.edu.tr; Tel: +90 2191010/2248; Fax:+903462191165

Table S1. Raman band assignment of ZIF-8 [1–3]

Frequency	Band assignment
74.2	νZn−N
168	νZn−N
273	νZn−N
686	Imidazole ring puckering
755	C=N ring rotation, δN−H
833	C−H ring rotation (C4−C5)
950	C−H ring rotation (C2−H)
1021	C−H ring rotation
1146	νC5−N
1180	νC−N+N−Hwag
1187	νC−N
1311	Ring expansion +N−Hwag
1384	δCH_3
1458	C−Hwag
1499	C2N3+C4N3+νC5N1+N-Hwag
1508	νC4−C5
2915	ν symmetric C−H(methyl)
2931	ν asymmetric C−H(methyl)
3110	νC−H(ar)
3131	νC−H(ar)

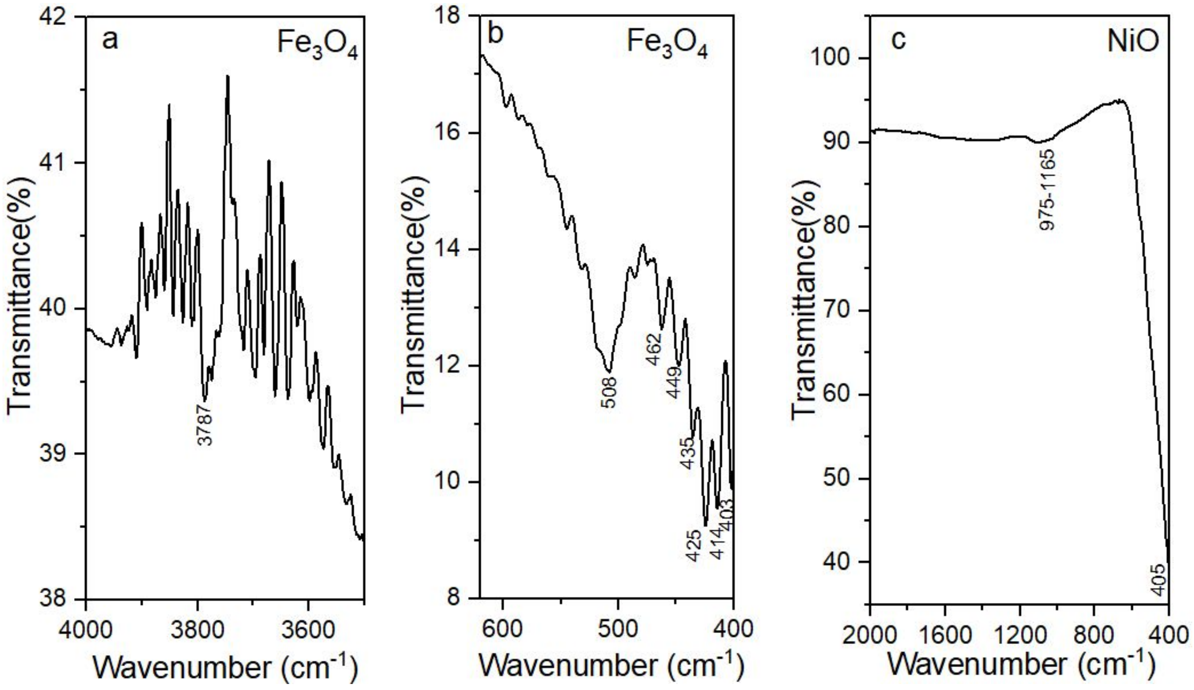

Figure S1. FTIR spectra of bare Fe_3O_4 nanoparticles (a and b) and bare NiO nanoparticules (c).

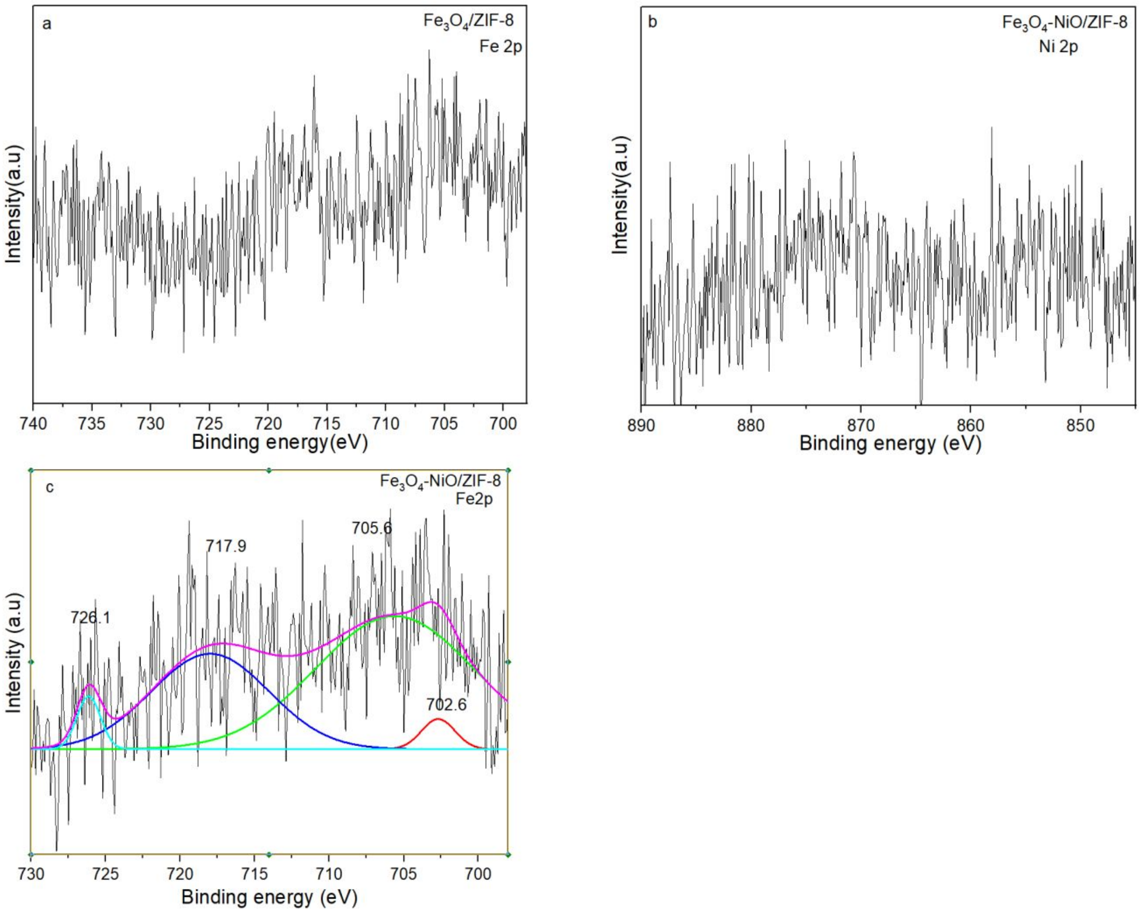

Figure S2. High-resolution XPS spectra of (a) Fe 2p Fe3O4/ZIF-8 (b) Ni 2p in Fe3O4-NiO/ZIF-8 (c) deconvoluted Fe 2p peaks for the Fe3O4-NiO/ZIF-8 nanocomposite.

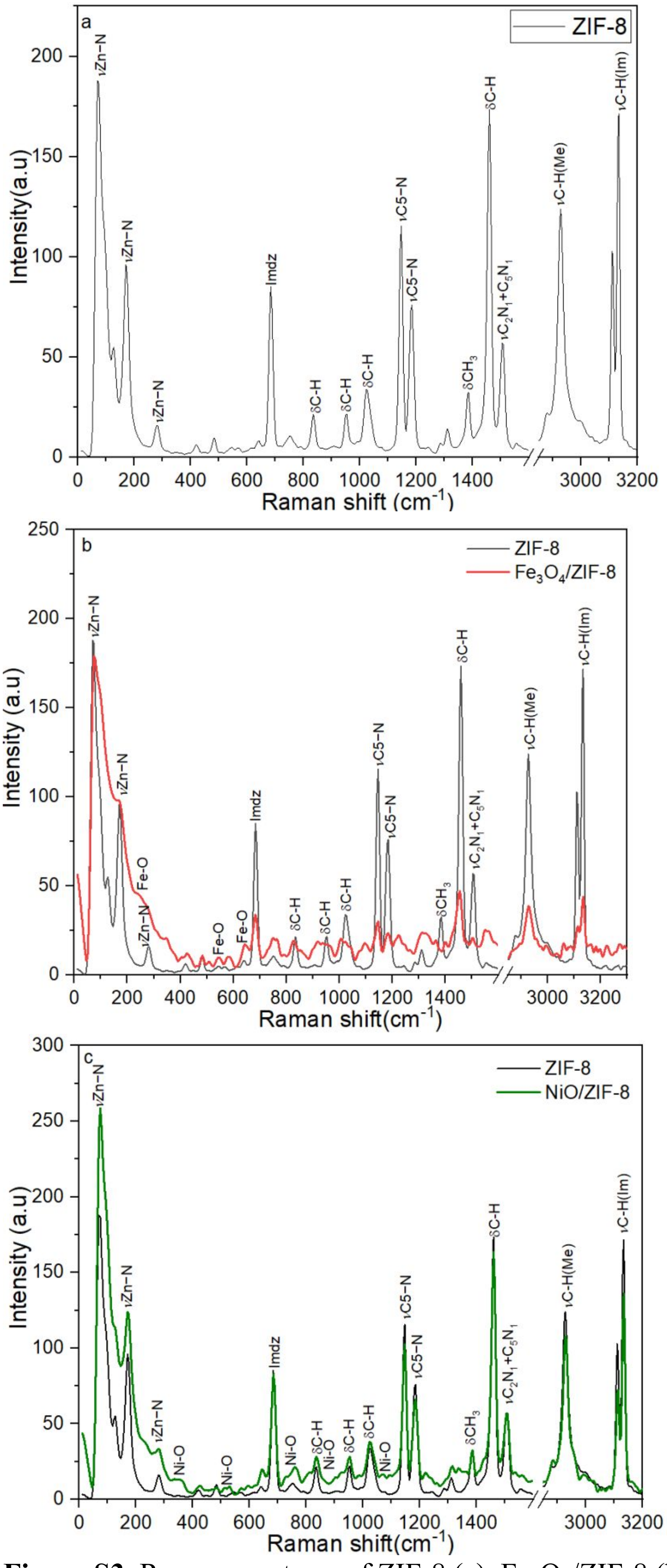

Figure S3. Raman spectrum of ZIF-8 (a), Fe_3O_4/ZIF-8 (b) and NiO/ZIF-8 (c)

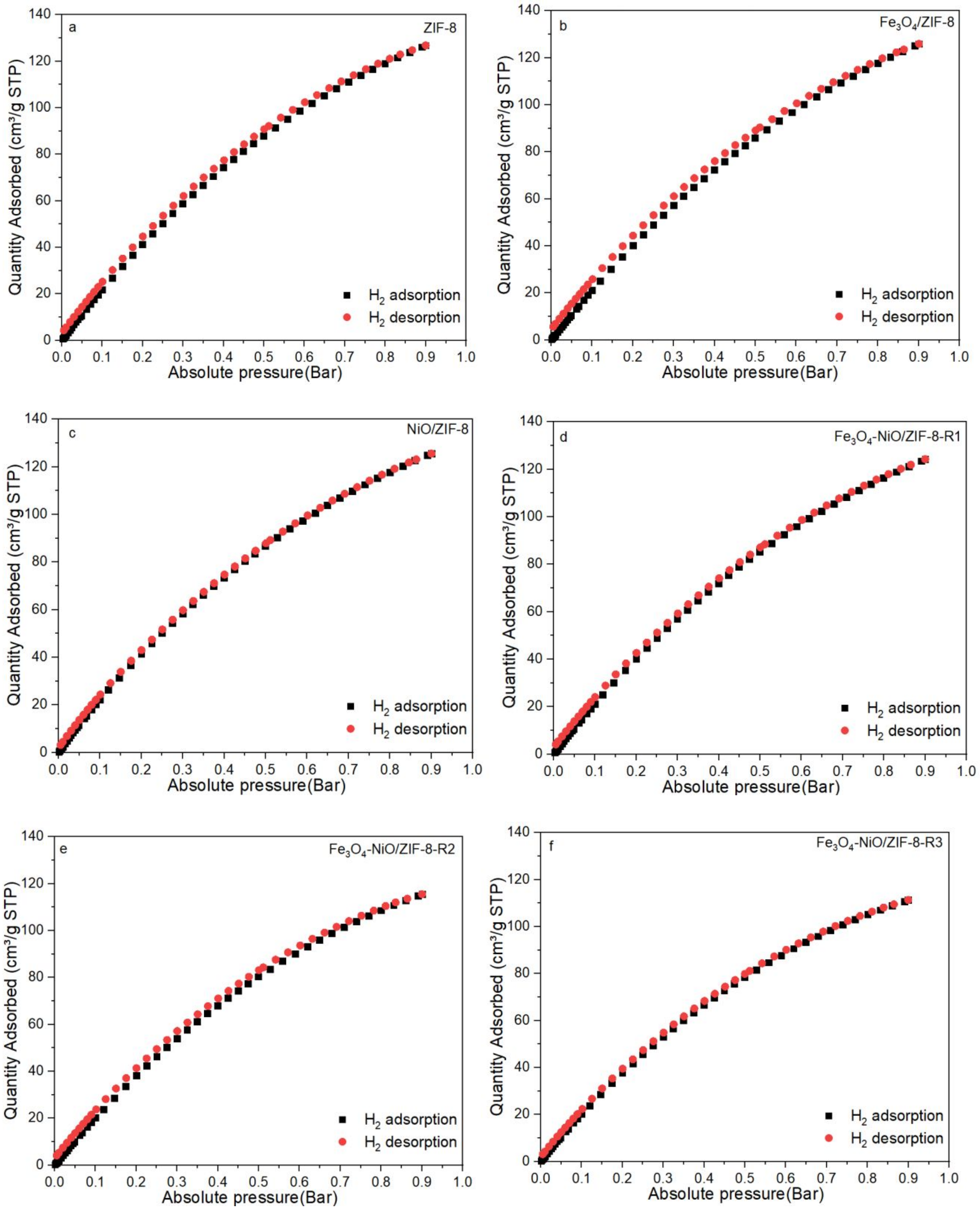

Figure S4. Hydrogen adsorption-desorption graph of **a)** ZIF-8, **b)** Fe_3O_4/ZIF-8, **c)** NiO/ZIF-8, **d)** Fe_3O_4-NiO/ZIF-8-R1, **e)** Fe_3O_4-NiO/ZIF-8-R2, **f)** Fe_3O_4-NiO/ZIF-8-R3

References

[1] Markham LM, Mayne LC, Hudson BS, Zgierski MZ. Resonance Raman studies of imidazole, imidazolium, and their derivatives: the effect of deuterium substitution. J Phys Chem 1993;97:10319–25.

[2] Carter DA, Pemberton JE. Raman spectroscopy and vibrational assignments of 1-and 2-methylimidazole. J Raman Spectrosc 1997;28:939–46.

[3] Mao C-J, Hu X-W, Song J-M, Niu H-L, Zhang S-Y. Synthesis of zinc 1-(2-pyridylazo)-2-naphthol (Zn (PAN) 2) nanobelts with nonlinear optical property. CrystEngComm 2012;14:6823–6.
